# Pilot Trial to Individualize the Dose of Follitropin Delta in Oocyte Donors: REKO15

**DOI:** 10.3390/jcm14124150

**Published:** 2025-06-11

**Authors:** Ignacio Cristóbal Quevedo, Manuel Piró, Sonia Matey, Abigail Álvarez, Mónica Toribio, Alfredo Guillén, Juan A. García-Velasco

**Affiliations:** 1 Department of Gynecology, Hospital Clínico San Carlos, 28040 Madrid, Spain; 2 IVIRMA Global Research Alliance, 28023 Madrid, Spain; 3 Instituto de Investigación Santiaria La Fe, 46026 Valencia, Spain; 4 Department of Gynecology and Obstetrics, Faculty of Medicine, Rey Juan Carlos University, 28933 Madrid, Spain

**Keywords:** follitropin delta, follitropin alfa, ovarian stimulation, oocyte donors

## Abstract

**Objectives:** This research aimed to study the effect of 15mcg/day of follitropin delta on normo-responding women. **Methods**: A single-center, open-label, matched case–control pilot trial was carried out from September 2021 to June 2022. In this trial, normo-responding oocyte donors were given 15 mcg/day of follitropin delta or 225 IU/day of follitropin alfa, as well as medroxyprogesterone acetate for pituitary suppression during the cycle. The main outcome measured was the number of oocytes retrieved. **Results**: A fixed dose of 15 mcg/day of follitropin delta for ovarian stimulation in normo-responders achieved an average of 17 oocytes retrieved. No differences were observed vs. 225 IU/day of follitropin alfa in the number of oocytes [17.8 ± 7.8 vs. 18.5 ± 7.7, respectively, *p* = 0.156], the number of metaphase II oocytes [13.5 ± 6.9 vs. 15 ± 6.3, *p* = 0.105], the fertilization rates (71.1% vs. 72.9%, *p* = 0.523), the number of usable blastocysts (4.9 ± 2.4 vs. 4.5 ± 2.5, *p* = 0.466), and the implantation rate (64% vs. 57%, *p* = 0.575). In the follitropin delta group, the duration of the stimulation was significantly shorter (9.4 ± 1.2 vs. 10.9 ± 1.2, *p* < 0.01), and the overall gonadotropin intake was lower. There were no clinically relevant differences between treatment groups regarding the safety profile. Global patient satisfaction with the follitropin delta ovarian stimulation was very high (7.7 ± 2.2). **Conclusions**: A daily dose of 15 mcg of follitropin delta may provide a similar ovarian response to 225 IU/day of follitropin alfa; aiming to retrieve 17 oocytes in normo-responders undergoing progestin-primed ovarian stimulation, it could reduce gonadotropin intake by reducing the duration of the stimulation cycle, with a possible high patient satisfaction level.

## 1. Introduction

Traditionally, the concept of a good response to exogenous gonadotropins in in vitro fertilization (IVF) was linked to retrieving a high enough number of oocytes to transfer one blastocyst and freeze the rest, but not so high that the patient was exposed to the risk of ovarian hyperstimulation syndrome (OHSS) [1]. Nowadays, OHSS risks can be almost completely prevented with a GnRH agonist trigger and vitrification [2], but this precludes a fresh transfer. Thus, the retrieval target of ovarian stimulation (OS) cycles should be individualized to match the desires and needs of each patient.

Personalized OS aimed at maximizing oocyte yield without compromising success or increasing OHSS risks has been evaluated in the last few years, and several prediction models and algorithms have been proposed [3,4]. The use of modern biomarkers such as anti-Müllerian hormone (AMH), combined with patient body weight, enabled the development of an easy-to-use algorithm that targets 8 to 14 oocytes with follitropin delta [5], the first recombinant FSH (rFSH) derived from a human cell line. Extensive trials worldwide have shown that by using this unique validated algorithm, we can minimize hyper- and poor-responders, reduce gonadotropin intake, and reduce OHSS, with clinical outcomes that are comparable to the conventional approach in terms of live birth rates and cumulative live birth rates [5,6,7].

Follitropin delta is a recombinant form of FSH produced using a human cell line, which gives it unique properties compared to other rFSH products. Its distinct glycosylation pattern results in slower clearance from the body and a heightened ovarian response at equivalent biological doses. Notably, it is the first human-derived rFSH that enables individualized dosing. In contrast, follitropin alfa, also a recombinant FSH, is produced in a Chinese hamster ovary cell line. It was the earliest rFSH available on the market and serves as a benchmark for comparison with newer FSH treatments [8].

Previously published evidence showed that by increasing the rFSH dose, patients produced more oocytes, but not more blastocysts or good-quality blastocysts [9]; however, Fanton et al. [10] recently showed that this might not be the case, as did a very extensive retrospective analysis of the database of the Society for Assisted Reproductive Technology. In fact, they clearly demonstrated that by increasing the ovarian response to gonadotropins, more metaphase II oocytes (MII) are obtained, without compromising the blastulation rate, even with extreme responses [10].

Although an extreme ovarian response is far from what we seek, as it would put patients at risk not only of OHSS but also of ovarian torsion, hemorrhage, and thromboembolic events [11], it does seem relevant to go beyond 8–14 oocytes as a target in certain OS protocols that do not include a fresh transfer.

A higher number of oocytes is likely to be beneficial for patients who wish to have more than one child, those undergoing preimplantation genetic testing for aneuploidies (PGT-A), those who opt for planned oocyte cryopreservation, or oocyte donors. In all these situations, patients may benefit from a higher rFSH dose, resulting in more oocytes.

Traditionally, FSH dosing was estimated in international units (IUs), based on the Steelman–Pohley assay [12], far less precise than rFSH products, which are filled by mass. But even today, filled-by-mass rFSH is converted into IU, as clinicians feel more comfortable using this measurement unit. In fact, 225 IU of rFSH alfa is routinely used in oocyte donation programs.

Follitropin delta provides a higher ovarian response in humans when administered not only in equal units of biological activity, as in the rat in vivo Steelman–Pohley assay [13], but also in the same microgram weight dose, as determined in a study [9,14] that established that a daily dose of 10 mcg of follitropin delta provides a similar ovarian response to 150 IU/day of follitropin alfa. Applying this equivalence factor, it is possible to extrapolate that 15 mcg of follitropin delta would be expected to provide a comparable ovarian response to 225 IU of follitropin alfa.

Our study aimed to evaluate the effects of a higher dose of follitropin delta in normo-responding patients [15] within a progestin-primed ovarian stimulation (PPOS) protocol, comparing the ovarian response to that achieved with the standard dose of 225 IU/day of follitropin alfa. This comparison is of clinical relevance in the current context, where the use of GnRH agonists as final oocyte maturation triggers has allowed for enhanced safety by drastically reducing the risk of OHSS, while enabling the targeting of more robust ovarian responses.

Although the follitropin delta dosing algorithm was originally developed to minimize the risk of OHSS in protocols utilizing hCG as the trigger [14], there are no prior studies exploring its performance at higher doses within the new safety paradigm offered by GnRH agonists. Our research aims to address this knowledge gap by assessing whether a controlled increase in follitropin delta dose (15 mcg/day) can achieve an oocyte yield equivalent to that of conventional-dose follitropin alfa (225 IU/day) [14], targeting 17 oocytes while maintaining the safety profile characteristic of PPOS protocols.

## 2. Materials and Methods

### 2.1. Study Design and Population

This was a single-center, open-label, matched case–control pilot trial conducted on oocyte donors from 28 September 2021 to 16 June 2022. The study was carried out in accordance with the Declaration of Helsinki on good clinical practice, and University Hospital Puerta de Hierro’s Ethical Committee approval was obtained (Ref. #1901-MAD-013-JG). It was authorized by the Spanish Health Authority (AEMPS) and registered at ClinicalTrials.gov (NCT04778358). All women eligible for this study signed a written informed consent form at the time they were scheduled at the IVI Clinic.

Inclusion criteria: We recruited oocyte donors aged 18 to 35, with normal uteri and ovaries confirmed by pelvic ultrasound and at least 6 antral follicles in each ovary on the starting day of stimulation. They had to be undergoing their first or second oocyte donation cycle and had to have an AMH < 6 ng/mL and a body weight of 50–85 kg. Volunteers were required to sign an informed consent form before screening and to comply with the Spanish Law on Human Reproduction.

Exclusion criteria: suffering from polycystic ovary syndrome [16] or stage III/IV endometriosis [17]; having used hormonal contraceptives (oral or vaginal) or Estradiol Valerate for cycle synchronization in the cycle prior to inclusion; having a family history of hereditary or chromosomal diseases or an abnormal karyotype; having tested positive for any sexually transmitted diseases as specified by the Spanish Law.

For comparisons, we selected a similar group of patients as controls (two controls per case) from our database. Patients had consented to the use of their anonymized data. This group of patients received a conventional dose of follitropin alpha (225 IU in normal responder patients) during the same time period of the duration of the study and were matched for age and body weight.

### 2.2. Study Outcomes

The primary endpoint of this study was the number of oocytes retrieved after a fixed daily dose of 15 mcg of follitropin delta (Rekovelle, Ferring Pharmaceuticals, St-Prex, Switzerland) during the entire stimulation, aimed at obtaining 17 oocytes.

The secondary endpoints of the study were the number of MII oocytes recovered, the duration of the ovarian stimulation, the total number of blastocysts, the implantation rates, and the incidence of OHSS using the Golan classification [18].

Patient satisfaction with the infertility treatment was also evaluated using the EFESO questionnaire [19]. This contains 17 items where patients are asked to rate their level of satisfaction on a 5-point Likert-type scale (from “very high” to “very low”). The total score ranges from 0 (minimum satisfaction) to 68 (maximum satisfaction). Topics cover satisfaction with the information and care received, satisfaction with medication storage, transportation, preparation, ease of use, time required, interference with daily activities, duration of treatment, number of injections, and issues related to safety, such as the possibility of correcting errors or adverse reactions.

### 2.3. Ovarian Stimulation

A fixed daily dose of 15mcg of rFSH delta (Rekovelle, Ferring, Switzerland) was administered to the oocyte donors for OS using a pre-filled injection pen, while the historical matched control group was given a fixed daily dose of 225 IU of rFSH alfa (Gonal-F, Merck Serono, Darmstadt, Germany).

All patients, both in the study group as well as in the control group, were given medroxyprogesterone acetate 10 mg PO once a day (Progevera, Pfizer, New York, NY, USA) for pituitary suppression starting on day 1 of the OS—during the first 3 days of menses—until the day they were administered the GnRH agonist (Decapeptyl 0.1 mL, Ipsen Pharma, Paris, France).

The GnRH agonist was administered when at least three follicles reached a mean diameter of 17–18 mm. An ultrasound-guided oocyte retrieval was performed transvaginally 36 h after triggering.

Oocyte donors were clinically evaluated and interviewed regarding their level of satisfaction with the procedure 5 days after the oocyte retrieval.

### 2.4. Statistical Analysis

Quantitative variables were described using the mean and standard deviation, and qualitative variables using absolute values and proportions. For comparison purposes, the chi-square test was used for proportions and an analysis of variance for quantitative variables. All statistical analyses were performed using the SPSS v22.0 statistical package (IBM Corp., Armonk, NY, USA). The α-error was set at 5%, and all statistical comparisons were two-sided. No sample size calculation was performed, given the pivotal approach of the study, which focused on the general understanding of the topic and the identification of trends.

## 3. Results

### 3.1. Baseline Characteristics

**Figure 1:**
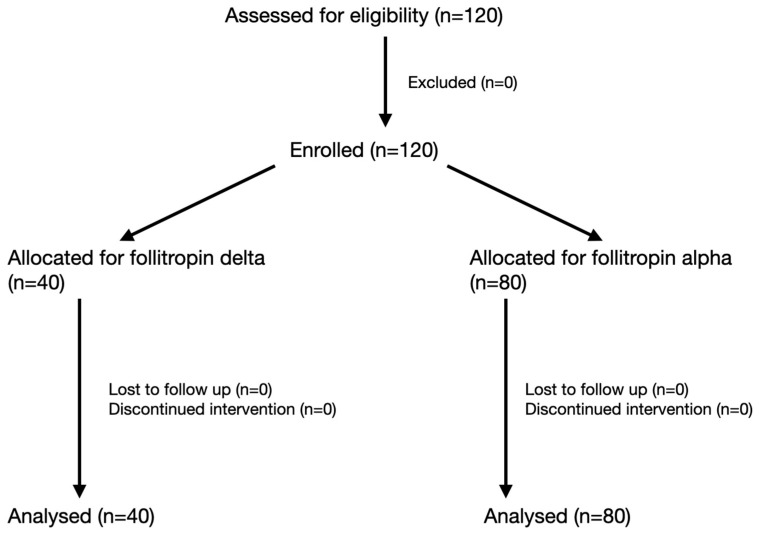
Flow of participants through the REKO15 trial.

A total of 120 patients were recruited and included in the study (Figure 1). The 40 (33.33%) patients included in the study group received 15 mcg/day of follitropin delta. They were matched for age, AMH, and body weight with the other 80 (66.67%) included in the historical control group, who received 225 IU/day of follitropin alfa. The demographic and clinical data of both arms are summarized in Table 1. Baseline characteristics were comparable in both arms, except for serum AMH levels, which were significantly lower in the follitropin delta group (19.3 ± 8.7 vs. 25.7 ± 8.9 pmol/mL, *p* = 0.001).

### 3.2. Stimulation Protocol

Data regarding ovarian stimulation, such as the number of oocytes retrieved, the number of MII oocytes, the days of stimulation, the fertilization and implantation rates, the number of blastocysts available, and the incidence of OHSS, are summarized in Table 2.

Regarding the primary outcome, 100% of the donors underwent an oocyte pick-up with a mean number of 17.8 ± 7.8 oocytes retrieved in the follitropin delta group. No differences were observed versus the follitropin alfa group [17.8 ± 7.8 vs. 18.5 ± 7.7, *p* = 0.156].

Secondary outcomes, such as the number of MII oocytes recovered [13.5 ± 6.9 vs. 15 ± 6.3, *p =* 0.105], the fertilization rates (71.1 ± 14.7% vs. 72.9 ± 16.8%, *p* = 0.523) or the number of usable blastocysts (4.9 ± 2.4 vs. 4.5 ± 2.5, *p* = 0.466), were comparable between cases and controls. As expected, no differences were found between treatment groups in implantation rate (64% vs. 57%, *p* = 0.575).

We observed significantly fewer days of stimulation in favor of follitropin delta (9.4 ± 1.2 vs. 10.9 ± 1.2 days, *p* < 0.01). The mean total dose of follitropin delta administered was 141mcg, whereas the follitropin alfa group received 2475 IU: by reducing the duration of OS, we reduced the total intake of follitropin delta.

### 3.3. Safety Outcomes

There were no clinically relevant differences between treatment groups regarding the safety profile. A comparable number of patients in both groups (21.6% vs. 22.8%, *p* = 0.532) experienced a hyper-response (>25 MII oocytes). Regarding the incidence of OHSS, no statistical differences were observed between cases and controls (2.5% vs. 3%, *p* = 0.719); all OHSS cases were mild, as no moderate/severe OHSS was described. There were no differences in other adverse events between the two treatment groups (headache, pelvic pain, pelvic discomfort).

Concerning the level of satisfaction in the follitropin delta group, which was rated from “very high” to “very low” using the EFESO questionnaire [19], the results of the survey showed a high level of satisfaction regarding information on how to prepare the medication, injection ease, number of injections received, dose precision in injected medication, and interference of the treatment with daily activity. On a scale of 0 (no pain) to 10 (unbearable pain), patients rated injection site pain at 2.2 ± 1.7 and total pain during the cycle at 3.1 ± 1.9. Global patient satisfaction with the ovarian stimulation cycle was 7.7 ± 2.2.

## 4. Discussion

Arce et al. [9] were able to determine the daily follitropin delta dose (mcg) that provided the same biological response as 150 IU/day of follitropin alfa in terms of ovarian response, taking into account not only the number of oocytes retrieved, but also other FSH-related pharmacodynamic parameters, such as follicular development and ovarian hormone response [9]. In our initial study, we observed that administering 15 mcg/day of rFSH delta to normo-responding women achieves a comparable ovarian response to that using a standard dose of 225 IU/day of rFSH alfa, targeting 17 oocytes in a PPOS protocol. Although the converted doses of follitropin delta and alfa appear similar, the unique glycosylation and lower clearance of follitropin delta [8] could enhance its biological activity and, consequently, its ovarian response compared to follitropin alfa. Although the two groups were initially matched, a statistically significant difference in AMH levels was observed at the end of the study, with lower values in the study group. However, it is important to note that both groups remained within the high-AMH range (AMH > 15 pmol/mL), which is associated with a strong response to ovarian stimulation. From a clinical perspective, this difference did not translate into meaningful variations in oocyte yield or in the incidence of OHSS, as these outcomes were comparable between groups. This suggests that above a certain AMH threshold, differences in ovarian reserve may have only subtle effects on the outcomes of ovarian stimulation.

Interestingly, in this trial, patients treated with follitropin delta seemed to require a lower number of days of OS prior to OPU, although further studies should be conducted. This finding is in line with a previous large, randomized, controlled trial that compared the efficacy and safety of follitropin delta in its individualized fixed-dose regimen versus follitropin alfa in a conventional adjustable dosing regimen in Asian women (9.2 ± 1.9 vs. 8.7 ± 1.6) [20]. While this finding could be biologically plausible, as follitropin delta has a closer structure to endogenous FSH than follitropin alpha [21], other studies [5,7,22] did not observe any differences.

In our study, we observed similar trends in the number of follicles >12mm measured by ultrasound and in the number of mature oocytes retrieved between the two groups. Nyboe Andersen et al. [5] did not find differences in the number of oocytes retrieved (10.0 ± 5.6 vs. 10.4 ± 6.5) or the number of blastocysts (3.3 ± 2.8 vs. 3.5 ± 3.2). Interestingly, Arce et al. [9] showed a positive dose–response relationship between the follitropin delta dose and the number of oocytes in a limited range of patients, although this did not translate into an increase in the number of blastocysts. However, recent data from a much larger sample analyzing 500,000 cycles showed a linear relationship between ovarian response, mature oocytes, and blastocyst formation [10], challenging the concept that beyond a certain threshold, oocytes recovered from atresia would not make good-quality blastocysts. Therefore, our results may be in accordance with the recent literature.

The routine use of an agonist trigger was found to be associated with a reduced incidence of severe ovarian hyperstimulation syndrome (OHSS) in the study participants. Mild OHSS was observed in both arms, with comparable frequencies suggesting a trend. In fact, when >15 oocytes are retrieved, an increase in abdominal discomfort, measured using a visual analog scale, is perceived in most patients [22]. In our group of patients, we observed a 2.5–3% incidence of mild OHSS, comparable to that described in larger studies such as the ESTHER-1 [5], or in studies with other rFSH molecules [23,24].

In the last few years, GnRH antagonist pituitary suppression has been replaced by progesterone, an approach that can be used when an embryo transfer is not planned in the same cycle, as is the case with oocyte donors. Progestin-primed ovarian stimulation (PPOS) has shown similar results to GnRH antagonist cycles in terms of the number of MII obtained, blastulation rates, euploidy rates, and ongoing pregnancy rates [25,26]. It is a patient-friendly protocol, as the medication is oral and significantly cheaper. This study has observed that, as expected, PPOS combined with follitropin delta appears to offer similar results to GnRH antagonist cycles.

Finally, we used the EFESO questionnaire [19] to measure our patients’ level of satisfaction, which is a fundamental part of the patient’s journey in fertility treatment and allows us to go one step beyond success rates. We observed that women found the treatment to be easy to prepare and inject, describing local pain at the injection site as low, with high global patient satisfaction. Evaluating patients’ healthcare experiences and needs is an essential element when assessing care quality; however, we know very little about patient preferences in OS treatments. Research is therefore needed if we want to incorporate these preferences into our healthcare policies and potentially improve the effectiveness of our treatments by enhancing patient satisfaction and treatment adherence [27]. It may also help healthcare staff to better meet patient needs, wishes, and priorities [28]. Individualized counseling is crucial, as patient profiles and expectations differ broadly [29].

The principal strength of this study is the evaluation of a higher dose of follitropin delta as an off-label use, which offers new possibilities for OS in our patients. Furthermore, the incorporation of patient satisfaction levels represents a novel approach that has the potential to enhance healthcare in the future. Nevertheless, it is important to acknowledge the limitations of this study. As a pilot study, the limited sample size restricts the generalizability of the findings. The retrospective nature of the control group data introduces the potential for bias due to inaccuracies and the absence of certain information, such as patient satisfaction. Moreover, the utilization of historical controls may result in the introduction of biases associated with alterations in clinical practice over time, although controls were selected during the same time period of the study. The absence of randomization increases the risk of selection bias and confounding factors, and there is a possibility of unmeasured variables influencing our results. These limitations highlight the preliminary nature of our findings and emphasize the need for larger, randomized controlled trials to validate our observations. Despite these constraints, our study offers valuable insights that can facilitate future research on higher doses of follitropin delta in OS protocols.

## 5. Conclusions

The 15 mcg/day dose of follitropin delta appears to show potential in approaching our goal of 17 oocytes, in what seems to be a patient-friendly ovarian stimulation protocol. Preliminary findings suggest the possibility of targeting a slightly higher number of blastocysts in women planning for fertility preservation or a large family, in oocyte donors, and in all patients on a pre-planned segmented IVF cycle aiming for PGT-A. While observations are encouraging, further research is needed to confirm whether this approach can consistently maintain favorable outcomes.

## Figures and Tables

**Table 1:** Demographic and clinical data for both groups. n = total number of patients; AMH = anti-Müllerian hormone; * = significant difference.

	F. Delta 15 mcg	F. Alfa 225 IU	*p* Value
**N**	40	80	
**Age (years)**	26.3 ± 4.2	26.4 ± 4.2	0.920
**Body weight (kg)**	63.4 ± 9.1	63.9 ± 8.9	0.775
**AMH (pmol/L)**	19.3 ± 8.7	25.7 ± 8.9	**0.001** *

**Table 2:** Ovarian stimulation cycle outcomes. n = total number; OS = ovarian stimulation; OHSS = ovarian hyperstimulation syndrome; FR = fertilization rate; IR = implantation rate; MII = metaphase II oocytes; * = significant difference.

	F. Delta 15 mcg	F. Alfa 225 IU	*p* Value
**O** **ocytes**	17.8 ± 7.8	18.5 ± 7.7	0.156
**MII**	13.5 ± 6.9	15 ± 6.3	0.105
**>25 MII**	21.6%	22.8%	0.532
**Days of OS**	9.4 ± 1.2	10.9 ± 1.2	**0.001** *
**OHSS**	2.5%	3%	0.719
**FR %**	71.1 ± 14.7	72.9 ± 16.8	0.523
**N blastocysts**	4.9 ± 2.4	4.5 ± 2.5	0.466
**IR**	64%	57%	0.575

## Data Availability

The data that support the findings of this study are not publicly available due to privacy and ethical restrictions. Interested researchers may request access to the datasets by contacting the corresponding author via email.
